# Computational Causal Modeling of the Dynamic Biomarker Cascade in Alzheimer's Disease

**DOI:** 10.1155/2019/6216530

**Published:** 2019-02-03

**Authors:** Jeffrey R. Petrella, Wenrui Hao, Adithi Rao, P. Murali Doraiswamy

**Affiliations:** ^1^Department of Radiology, Duke University Health System, Durham, NC, USA; ^2^Department of Mathematics, Penn State University, State College, PA, USA; ^3^Department of Psychiatry, Duke University Health System, Durham, NC, USA

## Abstract

**Background:**

Alzheimer's disease (AD) is a major public health concern, and there is an urgent need to better understand its complex biology and develop effective therapies. AD progression can be tracked in patients through validated imaging and spinal fluid biomarkers of pathology and neuronal loss. We still, however, lack a coherent quantitative model that explains how these biomarkers interact and evolve over time. Such a model could potentially help identify the major drivers of disease in individual patients and simulate response to therapy prior to entry in clinical trials. A current theory of AD biomarker progression, known as the dynamic biomarker cascade model, hypothesizes AD biomarkers evolve in a sequential but temporally overlapping manner. A computational model incorporating assumptions about the underlying biology of this theory and its variations would be useful to test and refine its accuracy with longitudinal biomarker data from clinical trials.

**Methods:**

We implemented a causal model to simulate time-dependent biomarker data under the descriptive assumptions of the dynamic biomarker cascade theory. We modeled pathologic biomarkers (beta-amyloid and tau), neuronal loss biomarkers, and cognitive impairment as nonlinear first-order ordinary differential equations (ODEs) to include amyloid-dependent and nondependent neurodegenerative cascades. We tested the feasibility of the model by adjusting its parameters to simulate three specific natural history scenarios in early-onset autosomal dominant AD and late-onset AD and determine whether computed biomarker trajectories agreed with current assumptions of AD biomarker progression. We also simulated the effects of antiamyloid therapy in late-onset AD.

**Results:**

The computational model of early-onset AD demonstrated the initial appearance of amyloid, followed by biomarkers of tau and neurodegeneration and the onset of cognitive decline based on cognitive reserve, as predicted by the prior literature. Similarly, the late-onset AD computational models demonstrated the first appearance of amyloid or nonamyloid-related tauopathy, depending on the magnitude of comorbid pathology, and also closely matched the biomarker cascades predicted by the prior literature. Forward simulation of antiamyloid therapy in symptomatic late-onset AD failed to demonstrate any slowing in progression of cognitive decline, consistent with prior failed clinical trials in symptomatic patients.

**Conclusions:**

We have developed and computationally implemented a mathematical causal model of the dynamic biomarker cascade theory in AD. We demonstrate the feasibility of this model by simulating biomarker evolution and cognitive decline in early- and late-onset natural history scenarios, as well as in a treatment scenario targeted at core AD pathology. Models resulting from this causal approach can be further developed and refined using patient data from longitudinal biomarker studies and may in the future play a key role in personalizing approaches to treatment.

## 1. Introduction

Alzheimer's disease (AD), one of the leading public health priorities in the U.S., is projected to affect over 15 million people by 2050. The high failure rate of clinical drug trials over the past decade is in large part rooted in an incomplete understanding of its complex causal mechanisms [1]. Genetic pathway analyses implicate over 1000 different molecular species and over 30 metabolic pathways in the pathophysiology of AD, including amyloid and tau proteinopathies, inflammation, microglial activation, alterations in signaling pathways, cholesterol metabolism, and cholinergic function [2]. It is therefore likely that AD is not a single disease, but a common end-stage pathway resulting from multiple interacting etiologies. Effective treatment will likely require a personalized medicine approach to track disease progression, determine the major pathophysiologic drivers, and tailor an appropriate therapy.

AD progression can be tracked in patients, from presymptomatic to late-stage disease, through several validated biomarkers. These are biomarkers of AD core pathology (cerebrospinal fluid and PET scan markers of beta-amyloid and tau proteins) and biomarkers of neuronal loss (FDG-PET and volumetric MR imaging). Data from Alzheimer's Disease Neuroimaging Initiative (ADNI), and other naturalistic studies, have led to a hypothetical model of disease progression known as the dynamic AD biomarker cascade theory [3], which hypothesizes that AD biomarkers evolve in a sequential but temporally overlapping manner. According to the hypothesis, amyloid pathology is an early event, leading to tau pathology, followed by neuronal loss and cognitive decline. Additional refinements of the model have been proposed to make it more generalizable to community-based aging populations, including the addition of suspected nonamyloid pathology (SNAP) (e.g., cerebrovascular disease, age-related changes, and non-AD tauopathies), as well as the concept of cognitive reserve (e.g., protective factors such as genetics or education), both of which could influence the variability in the onset of biomarkers and cognitive decline [4]. Although this theoretical model has been operationalized into a categorical scheme for classifying patients, the system remains descriptive and makes no assumptions regarding putative causal relationships among biomarkers [5]. Understanding how these biomarkers interact, evolve over time, and result in cognitive expression of disease will be essential to harness them in a personalized medicine approach to AD diagnosis and treatment. Given the complexity of AD, a rigorous mathematical and computational modeling approach, such as that offered by systems biology, will be a critical component.

The tools of systems biology may be used to incorporate clinical biomarkers of disease progression into a computational model to determine the major pathoetiologic drivers of disease in individual patients and help simulate the effects of potential interventions. One modeling approach, known as causal modeling, refers to an explicitly formulated mathematical description of the biological phenomena of interest, based on existing knowledge, in terms of cause and effect relationships. This is in contrast to correlative models which merely describe statistical associations between variables without regarding to the mechanism driving the phenomena under investigation. Our goal was to construct and test the feasibility of an initial computational causal model (CCM) of AD biomarker progression, based on the updated dynamic AD biomarker cascade theory [4, 6]. This would enable the theory to be tested rigorously with existing data and further refined as new data become available.

## 2. Methods

**Figure 1:**
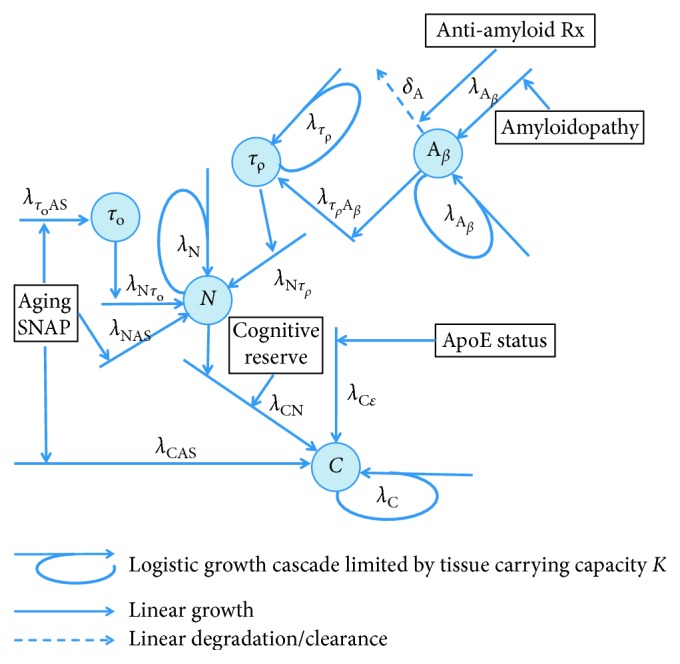
Diagram depicting causal modeling implementation of the biomarker cascade model in Alzheimer's disease [4, 6]. Blue circles represent biomarker quantities. *Α*_*β*_ represents the amyloid pathology. Its initial value determines that during the lifespan the amyloid cascade begins. *τ*_*ρ*_ represents the amyloid-related tau pathology (p-tau). *τ*_*0*_ represents the age-related and/or suspected non-Alzheimer pathology- (SNAP-) related tauopathy. *N* represents the neuronal dysfunction/loss. *C* represents the cognitive impairment. *λ* values are the growth rate constants, and *δ* a degradation/clearance rate constant. Amyloidopathy, aging, SNAP (suspected non-Alzheimer's pathology), ApoE status, and cognitive reserve are the constants that modify the onset of the growth cascades. Antiamyloid therapy is a function of time. The descriptions of all variables and the parameters are listed in Tables 1and 2, respectively (© 2017-2018 Duke University. All Rights Reserved).

For the construction of the causal model, assumptions about biomarker relationships and temporal course were drawn from the prior literature [3, 4, 6]. We tested whether the CCM would lead to the predicted biomarker trajectories described in the literature and whether it would predict failed outcome of antiamyloid therapy started late in the disease course [7].  Figure 1 shows the variables and their relationships in the computational model.

### 2.1. Computational Model Construction

We implemented the above causal model, using the ordinary differential equation (ODE) toolbox in MATLAB (Mathworks®, Natwick, MA), as the system of nonlinear first-order ODEs to include amyloid-dependent and nondependent neurodegenerative cascades. The amyloid-dependent cascade is initiated by amyloid beta, *A*_*β*_, and mediated via phosphorylated tau, *τ*_*ρ*_. The nonamyloid-dependent cascades are initiated by comorbidities, e.g., aging and/or suspected non-Alzheimer pathology (SNAP), either directly or indirectly via nonamyloid-dependent tauopathy, *τ*_*o*_. Initiation of cognitive decline, *C*, is directly determined by neurodegeneration, comorbidities, genetic factors, and cognitive reserve. The equations are as follows:

(1) $$\begin{array}{c} \frac{dA_{\beta }}{dt}=\;\lambda_{A_{\beta }}A_{\beta }\left({Κ}_{A_{\beta }}-A_{\beta }\right)+\lambda_{A_{\beta }A_{0}}A_{o}-\delta_{A_{\beta }}A_{\text{Rx}}\left(t\right),\\ \frac{d\tau_{\rho }}{dt}=\;\lambda_{\tau_{\rho }A_{\beta }}A_{\beta }+\lambda_{\tau_{\rho }}\tau_{\rho }\left({Κ}_{\tau_{\rho }}-\tau_{\rho }\right),\\ \frac{d\tau }{dt}=\;\lambda_{\tau_{\rho }A_{\beta }}A_{\beta }+\lambda_{\tau_{\rho }}\tau_{\rho }\left({Κ}_{\tau_{\rho }}-\tau_{\rho }\right)+\lambda_{\tau_{o}\text{AS}}\text{AS},\\ \frac{dN}{dt}=\;\lambda_{N\tau_{o}}\left(\tau -\tau_{\rho }\right)+\;\lambda_{N\tau_{\rho }}\tau_{\rho }+\lambda_{N}N\left({Κ}_{N}-N\right)+\lambda_{N\text{AS}}\text{AS},\\ \frac{dC}{dt}=\;\lambda_{CN}NR+\lambda_{C}C\left({Κ}_{C}-C\right)+\lambda_{C\text{AS}}\text{AS}+\lambda_{C\varepsilon }\varepsilon , \end{array}$$

where *A*_*β*_ represents the amyloid pathology; *τ*_*ρ*_ represents the amyloid-related tau pathology (p-tau);  *τ* represents the total tau pathology, defined as the sum of *τ*_*ρ*_ and *τ*_*0*_, where *τ*_*0*_ represents the age-related and/or SNAP-related tauopathy; *N* represents the neuronal dysfunction/loss; and *C* represents the cognitive impairment. *τ*_*0*_, rather than *τ*_*ρ*_, was explicitly modeled because it can be directly measured via assay. *λ* defines the numerous rate constants. *λ*_*A*_*β*__, *λ*_*τ*_*ρ*__, *λ*_*N*_, and *λ*_*C*_ reflect the logistic growth rates of the various biomarker cascades. The remaining rate constants reflect linear growth rates of the biomarkers and determine the influence of various factors on the time-of-onset of the subsequent biomarker cascades. *λ*_*CN*_, for example, is a rate constant that reflects the influence of neurodegeneration on cognitive decline, which is modified by cognitive reserve, for example, education level. This, along with comorbid pathologies and genetic risk alleles, determines the age of onset of the cognitive decline cascade. *δ*_*A*_*β*__ represents the degradation rate constant for *A*_*β*_ and, in this model, mediates the effects of antiamyloid therapy. *A*_*o*_ represents amyloidopathy, *A*_Rx_ (*t*) represents the time-dependent function for antiamyloid therapy, AS represents aging and/or SNAP, *R* represents cognitive reserve, and *ε* represents the ApoE allele status. The descriptions of all variables and the parameters are listed in Tables 1 and 2, respectively.

The additional assumptions of the model are as follows. (1) Biomarker cascade growth is implemented via a logistic growth model with carrying capacity *K*. *K* is adjusted using a least squares minimization procedure, so all biomarkers achieve the maximal level of 1 at the age of 100 years. This is done to configure biomarker curves in a sigmoidal shape with a progressively steeper slope in the right-hand tail for later changing biomarkers, as described in the hypothetical model. (2) At time *t* = 0, *A*_*β*_ is set to a very small number. This is done to initiate the amyloid cascade sometime during the lifespan, even in the absence of amyloidopathy. For simplicity, amyloidopathy *A*_*o*_, is set to zero in all models, and a slightly larger initial value of *A*_*β*_ is used for the early-onset model, whereas a slightly smaller value is used in the late-onset models. All other biomarker initial values are set to zero, except for total tau in the late onset models, which is set to the minimum biomarker level on the graphs. (3) The minimal biomarker level on the graphs is set to 0.05 to allow for different onset delays for the sigmoidal-shaped biomarker curves that depend upon both biology and biomarker sensitivity. Minimal detection level is set to 0.15. (4) Amyloidopathy, SNAP, aging, and ApoE status are constants across the age span that add linearly to the growth rate of the biomarkers and cause earlier initiation of the amyloid, tau, neurodegenerative, and/or cognitive decline cascades. (5) Cognitive reserve is a constant that modifies the effect of neuronal degeneration on the onset of cognitive decline. A lower value is used in the low-risk group, and a higher value in the high-risk group. (6) Antiamyloid therapy, once initiated, is assumed to be maintained throughout the lifespan, and *A*_Rx_ (*t*) is simulated as a Heaviside step function, H[*n*], using the half maximum convention:

(2) $$\begin{array}{c} Η\left[n\right]=\left\{\begin{array}{c} 0,\;n<0,\\ \frac{1}{2},\;n=0,\\ 1,\;n>0, \end{array}\right. \end{array}$$

where *n* represents the age of initiation of therapy. In the case of antiamyloid therapy, carrying capacity, *K*, was determined for all biomarkers under the no-therapy condition, in a natural history context, with *δ*_*A*_*β*__ set to zero.  *δ*_*A*_*β*__ was then changed to positive number to simulate the effects of amyloid degradation, with fixed *K* values based on natural history. This was done to assure that the evolution of biomarkers in the pretherapy interval was in no way influenced by the administration of therapy later in the course of the disease.

To determine the feasibility of the CCM, we parameterized and tested four versions: (1) early-onset autosomal dominant AD, (2) late-onset amyloid-first AD, (3) late onset tau-first AD, and (4) antiamyloid therapy in late-onset amyloid-first AD. In the first three scenarios, the goal was to determine whether manipulating the CCM parameters in a physiological meaningful manner could reproduce biomarker trajectories that closely match those visually depicted in the literature. In the fourth scenario, we determine whether the model would predict the outcome of recently failed clinical trials of antiamyloid therapy administered in symptomatic late-onset AD [7].

## 3. Results

### 3.1. Computational Model of Early-Onset Autosomal Dominant AD

**Figure 2:**
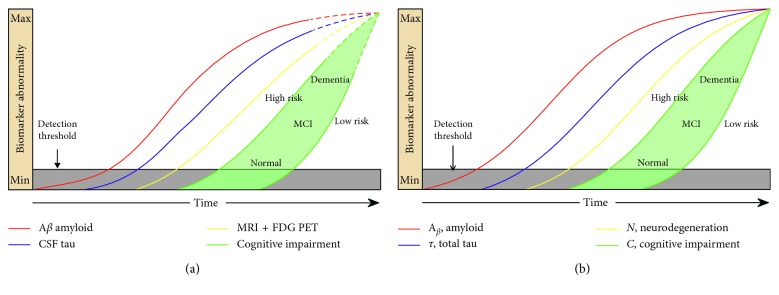
Model of early-onset, autosomal dominant AD. The red, blue, and yellow curves represent the evolution of amyloid, tau, and neuronal biomarker levels, respectively, over the course of the disease. The green curves represent cognition in two hypothetical high- and low-risk groups based on low and high cognitive reserve. Our CCM-generated curves (b) closely match the schematic model curves (a) (adapted from [6] with permission) from the prior literature.

Figure 2 illustrates that the cascade of early-onset familial AD derived from the prior literature [6] (Figure 2(a)) matches closely to the output generated by our DCM (Figure 2(b)). Specifically, they both demonstrate the initial appearance of amyloid, followed by tau and neurodegeneration, then followed by the onset of cognitive decline. It also shows how cognitive reserve could modify the cascade. Although tau in these figures represents total-tau, in this scenario, it is dominated by p-tau (amyloid-related tau). The model parameters are shown in Table 2.

### 3.2. Computational Model of Late-Onset Amyloid-First AD

**Figure 3:**
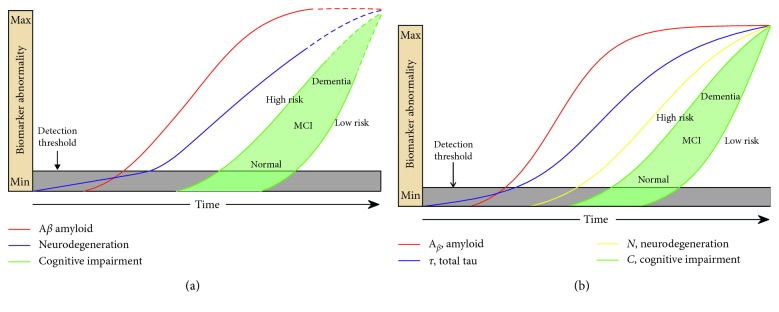
Model of late-onset, amyloid-first AD. The red, blue, and yellow curves represent the evolution of amyloid, tau, and neuronal biomarker levels, respectively, over the course of the disease. In (a), the blue and yellow lines are combined into a single purple line, per the original theory in which tau was considered a neurodegenerative marker. The green curves represent cognition in two hypothetical high- and low-risk groups, based on cognitive reserve. Our CCM-generated curves (b) match closely the pattern hypothesized in the literature ((a) is adapted from [6] with permission).

The late-onset AD CCM (Figure 3) output shows that amyloid appears first, followed by total tau and neurodegeneration. In this CCM, the arrival of amyloid is delayed compared to that in early-onset AD but reaches detection threshold prior to total tau. The CCM trajectories visually matched those predicted in the literature [6]. The model parameters are shown in Table 2.

### 3.3. Computational Model of Late-Onset Tau-First AD

**Figure 4:**
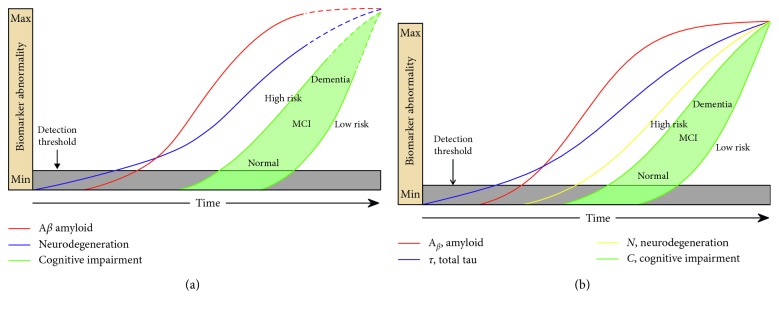
Model of late-onset tau-first AD. The red, blue, and yellow curves represent the evolution of amyloid, tau, and neuronal biomarker levels, respectively, over the course of the disease. In (a) (adapted from [6] with permission), the blue and yellow lines are combined into a single purple line, per the original theory in which tau was considered a neurodegenerative marker. The green curves represent cognition in two hypothetical high- and low-risk groups, based on cognitive reserve. The CCM (b) closely matches the trajectories proposed in the literature (a).

In this CCM (Figure 4), the arrival of total-tau precedes that of amyloid and initiates neurodegeneration, whereas the subsequent appearance of amyloid accelerates this process. Our CCM mimics a condition described in the literature as suspected nonamyloid pathology (SNAP) in some ways (absence of initial amyloid) but illustrates a mixed pathology concept, where amyloid and amyloid-related tau contribute to cognitive decline at later stages. The model parameters are shown in Table 2.

### 3.4. Computational Model of Antiamyloid Therapy Administered in Late-Onset Amyloid-First AD

**Figure 5:**
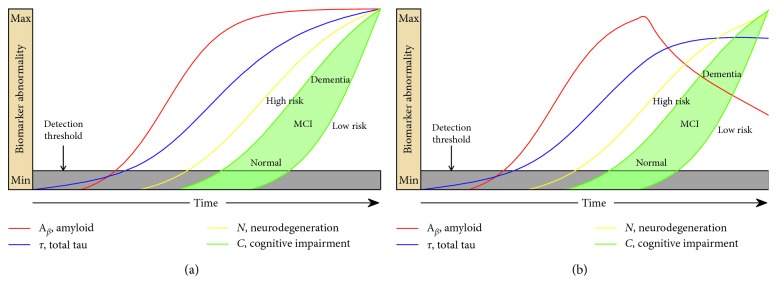
Model of anti-amyloid therapy in late-onset amyloid-first AD. (a) shows the untreated condition for comparison, reproduced from Figure 3(b). (b) shows the CCM simulation of the effect of antiamyloid therapy administered in AD after symptom onset. The red curve shows marked decline in brain amyloid levels, the blue line shows a small decrease in tau levels, and green lines show there is no significant effect on cognitive decline onset or rate, consistent with the many failed trials.

Figure 5 depicts the outcome of our CCM of antiamyloid therapy when given to amyloid-first late-onset AD dementia patients after symptom onset. This model output shows no benefit on the onset or slope of cognitive decline, despite the amyloid level dropping substantially from its peak. Tau levels drop marginally. The model mimics the results of recent failed antiamyloid therapy trials in probable AD dementia [7]. In this model, antiamyloid therapy would have to be given before a hypothetical tipping point to show benefits on cognition. The model parameters of Figure 5(b) are shown in Table 2.

## 4. Discussion

We have implemented a CCM that incorporates the three clinically available categories of biomarkers to track AD progression, amyloidopathy, tauopathy, and neurodegeneration. The model effectively simulates the temporal evolution of the biomarkers and their relation to cognitive decline as described in the previous literature [3, 4, 6], taking into account late verses early onset, the influence of aging and co-occurring non-AD-related brain pathology common in the elderly, and the concept of cognitive resilience to AD pathologic changes. In addition, we simulate the effects of a disease-modifying therapy given late in the disease course, after patients becoming symptomatic. This CCM was developed both as a means to test existing theories and as a new resource for the field that can be refined as our knowledge advances.

The hypothetical model of the AD pathological cascade, originally published in 2010 [3], and updated in 2013 [4], is based largely on cross-sectional biomarker data due to limited individual longitudinal biomarker data. It postulates a temporal evolution marker of amyloid pathology, tau pathology, and neurodegeneration, represented as sequential plots of biomarker abnormality over time, leading to cognitive impairment. Three different pathological and neuronal loss scenarios were considered, early-onset familial AD, late-onset amyloid-first AD, and late-onset tau-first AD [6]. We created and parameterized a CCM, based on assumptions of underlying biology inherent in the AD pathological cascade, to successfully simulate these three natural history scenarios. Our CCM of early-onset autosomal dominant AD closely matched the temporal order and shape of the biomarker trajectories in the literature schematic [3] and is also supported by empirical data from the longitudinal Dominantly Inherited Alzheimer Network (DIAN) study [8]. Our CCM of the late onset amyloid-first and tau-first models of AD also closely simulated the curves postulated in the literature [4]. Lastly, our simulation of antiamyloid therapy in symptomatic late-onset amyloid-first AD mimicked the negative findings from several failed clinical trials of antiamyloid therapies [7]. Of note, most of the disease modifying treatment trials in preclinical or in mild AD, recently completed and ongoing, target beta-amyloid or tau pathologies.

There are some limitations to our work. First, the hypothetical model [6] on which we built our CCM may not be entirely accurate. Although some aspects of the AD pathological cascade model, for example, the ordering and shape of the biomarker curves, have been validated using data-driven approaches [9, 10], the model continues to evolve as more natural history and clinical trial data becomes available. Second, we did not parameterize our models using actual patient biomarker data. Rather, our goal in this work was to construct and test the feasibility of a CCM that would best match the hypothetical biomarker trajectories proposed in the literature [6]. Subsequent goals will include adjusting parameters based on longitudinal data and iteratively refining the model itself as new knowledge becomes available. Third, our CCM is a simplified causal model of biomarkers interacting with each other, an abstraction that does not model the actual underlying cellular and molecular processes. Prior CCM efforts in AD have modeled the disease at a molecular and cellular level [11, 12] as well as at a whole brain, systems level using MRI and EEG data [13, 14]. There have been few prior CCM applications that have specifically focused on clinical AD biomarkers [13, 15], and only one has incorporated all three clinically available categories of biomarkers, amyloidopathy, tauopathy, and neurodegeneration [16]. Augmenting these efforts and overcoming the above limitations will require large real-world datasets of individual longitudinal biomarker trajectories across the cognitive continuum as well as integration of genomic, cellular, and biomarker knowledge. Such efforts are underway on an international scale [17–20].

A key strength of the CCM approach is that it allows for testing underlying causal assumptions in an integrated fashion, unlike other published correlative mathematical models of clinical biomarkers [9, 10, 21–27] that treat them independently or fit the data without considering its underlying causal structure. For example, several studies validated the temporal ordering of biomarkers, without attempting to explain the underlying disease mechanism by which this temporal ordering arises [9, 10, 26, 27]. Causal models allow for testing the effects of nonlinear interactions among multiple AD biomarkers and comorbid conditions that cannot be deduced by intuition alone, as well as for predicting response to single and combination therapies. A CCM can be implemented in a “forward” manner to simulate new data or in a “backward” manner, using a Bayesian inversion procedure, to infer the causal architecture of the system based on existing data. This approach has been applied extensively to reconstruct mechanistic models of brain function and disease, including AD, from electrophysiologic and imaging data [13, 15, 28]. Unlike descriptive models of disease, which can be become increasingly difficult to validate, particularly as datasets of biomarker trajectories become larger and more complex, CCM's can be easily scaled up to increasing degrees of complexity. It is our hope that once a CCM resource for clinical AD biomarkers is created, it will be parameterized based on data from patient studies, expanded and iteratively refined over time. Ultimately such a model would create a global resource for the field to translate existing knowledge, personalize care, and accelerate drug discovery for this devastating disorder [29].

## Figures and Tables

**Table 1:** Model variables.

Variable	Description
*A*_*β*_	Amyloid beta pathology
*τ*_*ρ*_	Amyloid-related phospho-tau pathology
*τ*_*o*_	SNAP-related tau pathology
*N*	Neuronal degeneration
*C*	Cognitive impairment

SNAP = suspected non-Alzheimer's pathology.

**Table 2:** Model parameters for early- and late-onset AD and antiamyloid therapy scenarios.

Parameter	Description	Early onset	Late-onset amyloid-first	Late-onset SNAP-first	Antiamyloid Rx (late-onset amyloid-first)
*A*_*β*0_	Amyloid beta pathology, initial value	0.05	0.01	0.01	0.01
*τ*_*ρ*0_	Tau (amyloid-related) pathology, initial value	0	0	0	0
*τ*_o0_	Tau (SNAP-related) pathology, initial value	0	0.05	0.05	0.05
*N*_0_	Neurodegeneration, initial value	0	0	0	0
*C*_0_	Cognitive impairment, initial value	0	0	0	0
*K*_Αβ_	Amyloid beta pathology, carrying capacity	1	1	1	1
*K*_τ*p*_	Tau (amyloid-related), carrying capacity	1	1	1	1
*K*_C_	Cognitive impairment, carrying capacity	1	1	1	1
*K*_*N*_	Neurodegeneration, carrying capacity	1	1	1	1
*λ*_Α*β*_	Amyloid cascade, growth rate	0.08	0.12	0.1	0.12
*λ*_Α*β*Α*Ο*_	Amyloid pathology from amyloidopathy, growth rate	0	0	0	0
*δ*_A*β*_	Amyloid degradation/clearance rate	0	0	0	0.04
*λ*_τ*pβ*_	Tau (amyloid-related) from amyloid, growth rate	0.025	0.025	0.025	0.025
*λ*_*τp*_	Tau (amyloid-related) pathological cascade, growth rate	0.05	0.05	0.05	0.05
*λ*_*τo*AS_	Tau (SNAP-related) path from aging/SNAP, growth rate	0.002	0.002	0.002	0.002
*λ*_*C*_	Cognitive impairment cascade, growth rate	0.05	0.05	0.05	0.05
*λ*_*CΝ*_	Cognitive impairment from neurodegeneration, growth rate	0.001	0.001	0.001	0.001
*λ*_*C*AS_	Cognitive impairment from aging/SNAP, growth rate	0	0	0	0
*λ*_*CΕ*_	Cognitive impairment from genetic risk to growth rate	0.001	0.001	0.001	0.001
*λ*_*Ν*_	Neurodegeneration cascade, growth rate	0.05	0.05	0.05	0.05
*λ*_*Ν*τ*p*_	Neurodegeneration from tau (amyloid-related), growth rate	0.025	0.025	0.025	0.025
*λ*_*ΝΤο*_	Neurodegeneration from tau (SNAP-related), growth rate	0.0075	0.0075	0.0075	0.0075
*λ*_*Ν*ΑS_	Neurodegeneration from aging/SNAP, growth rate	0	0	0	0
*A*_*O*_	Amyloidopathy	0	0	0	0
*A*_Rx_	Age of onset of antiamyloid therapy	0	0	0	65
AS	Aging, SNAP	0	1	2	1
*E*	ApoE allele genetic risk	0	0	0	0
*R*_1_	Cognitive reserve (low risk)	1	1	1	1
*R*_2_	Cognitive reserve (high risk)	25	25	25	25

## Data Availability

The data presented in this manuscript are based on simulations and therefore can be reproduced, given the equations, parameters, and descriptions in this article.
